# Uptake and correlates of cervical cancer screening among HIV-infected women attending HIV care in Uganda

**DOI:** 10.1080/16549716.2017.1380361

**Published:** 2017-10-16

**Authors:** Rhoda K. Wanyenze, John Baptist Bwanika, Jolly Beyeza-Kashesya, Shaban Mugerwa, Jim Arinaitwe, Joseph K. B. Matovu, Violet Gwokyalya, Dickson Kasozi, Justine Bukenya, Fred Makumbi

**Affiliations:** ^a^ Department of Disease Control and Environmental Health, Makerere University School of Public Health, Kampala, Uganda; ^b^ Department of Epidemiology and Statistics, Makerere University School of Public Health, Kampala, Uganda; ^c^ AIDS Control Program, Ministry of Health, Kampala, Uganda; ^d^ Global Fund Focal Coordination Office, Ministry of Health, Kampala, Uganda; ^e^ Department of Community Health, Makerere University School of Public Health, Kampala, Uganda

**Keywords:** Cervical cancer, cervical cancer screening, HIV-infected women

## Abstract

**Background**: Human immunodeficiency virus (HIV)-infected women are at high risk of cervical cancer.

**Objective**: This study assessed uptake and correlates of cervical screening among HIV-infected women in care in Uganda.

**Methods**: A nationally representative cross-sectional survey of HIV-infected women in care was conducted from August to November 2016. Structured interviews were conducted with 5198 women aged 15–49 years, from 245 HIV clinics. Knowledge and uptake of cervical screening and human papillomavirus (HPV) vaccination were determined. Correlates of cervical screening were assessed with modified Poisson regression to obtain prevalence ratios (PRs) using Stata version 12.0.

**Results**: Overall, 94.0% (*n *= 4858) had ever heard of cervical screening and 66% (*n *= 3732) knew a screening site. However, 47.4% (*n *= 2302) did not know the schedule for screening and 50% (*n *= 2409) did not know the symptoms of cervical cancer. One-third (33.7%; *n *= 1719) rated their risk of cervical cancer as low. Uptake of screening was 30.3% (*n *= 1561). Women who had never been screened cited lack of information (29.6%; *n *= 1059) and no time (25.5%; *n *= 913) as the main reasons. Increased likelihood of screening was associated with receipt of HIV care at a level II health center [adj. PR 1.89, 95% confidence interval (CI) 1.29–2.76] and private facilities (adj. PR 1.68, 95% CI 1.16–3.21), knowledge of cervical screening (adj. PR 2.19, 95% CI 1.78–2.70), where to go for screening (adj. PR 6.47, 95% CI 3.69–11.36), and low perception of risk (adj. PR 1.52, 95% CI 1.14–2.03). HPV vaccination was 2%.

**Conclusions**: Cervical screening and HPV vaccination uptake were very low among HIV-infected women in care in Uganda. Improved knowledge of cervical screening schedules and sites, and addressing fears and risk perception may increase uptake of cervical screening in this vulnerable population.

## Background

Cervical cancer is a major cause of death globally and the burden of disease is highest in low-income regions, especially sub-Saharan Africa (SSA) [1]. The incidence of cervical cancer in SSA, especially in Eastern and Western Africa, is the highest in the world. Countries in SSA generally have a high prevalence of human papillomavirus (HPV), the virus responsible for most cases of cervical cancer, and other risk factors including high human immunodeficiency virus (HIV) prevalence [2]. HIV-infected women have a higher risk of developing cervical cancer and faster disease progression [3,4]. In a study conducted among 2508 women in Rwanda, HPV prevalence was 34% and was highest among women aged ≤ 19 years. The prevalence of HPV and cytological abnormalities were significantly higher in HIV-positive than in HIV-negative women [5]. Similar findings have been reported in India, Malawi, Uganda, and other countries [6–9]. A study that evaluated cervical screening techniques in Uganda found that HIV-infected women had a higher prevalence of cervical intraepithelial neoplasia grade 2+ than uninfected women (12.9% vs 1.7%, respectively) [8].

Despite the heavy burden of disease, cervical cancer is one of the most easily preventable cancers among women. In 2014, the World Health Organization developed a comprehensive cervical cancer control guide, which includes: (1) primary prevention to reach girls aged 9–13 years with HPV vaccination to prevent HPV infection; (2) secondary prevention through cervical screening technologies (e.g. Papanikolaou smear, HPV testing, and visual inspection of the cervix with acetic acid followed by treatment of the detected precancerous lesions); and (3) tertiary prevention including cancer treatment and palliative care where treatment is no longer possible [1]. The implementation of this package has encountered challenges in low-income countries and especially in SSA.

Despite the reduction from three to two doses of HPV vaccine, which was intended to reduce costs and ease administration, uptake of HPV vaccine has remained low owing to cost and lack of knowledge [10]. Cervical cancer screening remains very low across several countries in SSA because of low levels of awareness, challenges with health-seeking behavior, and health system barriers [11]. A study in two districts in Eastern Uganda found that only 4.8% of women aged between 25 and 49 years had ever been screened for cervical cancer, and most had been screened owing to symptoms which they associated with cervical cancer [12]. Symptom-based screening contributes to delays in diagnosis. A study in Northern Uganda found that most women with cervical cancer were diagnosed at stages III (45%) and IV (21%) [13].

Improved communication with women to advance their knowledge is critical to increase access to all levels of cervical cancer prevention [1]. However, the level of knowledge of cervical cancer and screening varies across countries and within countries. Recent studies in Uganda show high levels of knowledge of risk factors, symptoms, and service options for the prevention and management of cervical cancer [14], while others show very low levels of knowledge [12]. Furthermore, there is a disconnect between high levels of knowledge and service uptake [15], and health system challenges abound [7,16]. For HIV-infected women, service uptake is hampered by limited integration of HIV and cervical cancer prevention and treatment services [17,18]. Where integration has happened, there is limited tracking of service uptake. Thus, large-scale studies exploring women’s knowledge and the integration and uptake of services are required to inform planning and monitoring trends in service uptake. In this cross-sectional study of 5198 HIV-infected women, we assessed the knowledge of cervical cancer screening, uptake of HPV vaccination, and cervical screening at 245 HIV clinics.

## Methods

Data were drawn from a quantitative national cross-sectional survey of HIV-infected women in care. The survey objectives were to assess the unmet need for family planning and determine the uptake of other reproductive health service including cervical cancer screening. The questions relating to cervical cancer included questions on knowledge and uptake of cervical cancer screening and HPV vaccination.

### Study sites

In total, 245 public and private HIV clinics across the five geographical regions in Uganda (Central, Northern, Eastern, Western, and Kampala) were included in the study. Kampala was taken as a separate region owing to its uniqueness as an entirely urban district and because it is the capital city of Uganda. The health facilities were selected from public and private facilities across various levels of service delivery; hospitals, and the lower level health centers (HCIV, HCIII, and HCII) with chronic HIV care clinics. The healthcare delivery system in Uganda is hierarchically organized from HCII, at the lowest level, to HCIV and district hospitals. Above the district hospitals are the regional referral and national referral hospitals.

### Sampling

A two-stage sampling process was used. In the first stage, a sampling frame with a list of accredited HIV care facilities in Uganda was used to randomly select an equal number of facilities in each region. Based on reports from the Ministry of Health, accredited facilities with at least 50 female clients registered in HIV care were selected. The second stage of sampling was selection of study participants at the facility level. All HIV-infected women aged 15–49 years who presented at the selected clinics for care on the interview days were registered on daily attendance sheets. Systematic sampling was then conducted to randomly select the required number of eligible women from the attendance list; the sampling interval varied across facilities based on the client volume. After sampling, a brief screening tool was used to assess eligibility, including age and those who were sexually active (had sexual contact at least once within 12 months). Eligible participants provided written informed consent before the interview was conducted.

### Sample size

The sample size calculation was based on unmet need for family planning as the main outcome, assuming *p* = 30% unmet need for family planning among HIV-positive women in care, 3.6% margin of error, 5% type I error rate, a design effect of 1.5, and a non-response rate of 10%. The overall sample size for the five geographical regions was thus 5185 (1037 respondents per region). Approximately 20 participants were selected from each facility except for the highest volume facilities in Kampala, with over 5000 clients in care, where 30–50 participants were selected per site. Kampala has fewer but larger volume dedicated HIV facilities compared to the other regions. Overall, participants were selected from 52 facilities in each region except for Kampala, where participants were drawn from 37 facilities.

### Data collection

Pretesting of tools was conducted in three health facilities outside the study districts to check the suitability of various aspects of the questionnaires such as the translation, skip procedures, and filtering questions; and modifications were made thereafter. All data collection tools were translated into the common languages of the selected regions. The fieldwork was conducted by a team of 25 experienced and fully trained and supervised interviewers. Data were collected between August and November 2016.

### Measures

Variables included sociodemographic characteristics, disclosure of HIV status to sexual partner, relationship status, HIV status of the partner, health status of the participant, HIV treatment status [antiretroviral therapy (ART) versus non-ART and duration on ART], and barriers to access (e.g. distance to the health facility where they were enrolled from). Respondents were also asked about their reproductive history, including the number of biological children, attendance at antenatal care (ANC), delivery at health facilities; and whether they had ever been screened for cervical cancer, how many times, and when they had last been screened. Women also answered a series of questions on cervical cancer knowledge, including questions on the symptoms and causes of cervical cancer. Those who had never been screened were asked why they had never been screened, while those who had been screened were asked about their experiences and whether they would undergo screening again. Women were also asked to rate their risk of cervical cancer in their lifetime on a scale of 1 (lowest) to 10 (highest). Finally, they were asked whether they had ever received HPV vaccination and how many times they received the vaccination. A facility checklist included a question on whether cervical screening was offered at the 245 facilities.

### Data management and analysis

Data were reviewed on a daily basis by the field data editors and supervisors, and weekly review meetings were held with the data collection teams to ensure quality. Trained data entrants carried out double-data entry. Descriptive analyses were conducted on the women’s sociodemographic characteristics, HIV diagnosis and treatment status, knowledge and uptake of cervical screening services, and partner characteristics, including their HIV status.

Prevalence ratios (PRs) were used as the measure of association between uptake of cervical cancer screening and women’s and facility characteristics. PRs were obtained via a modified Poisson regression model with robust standard errors, accounting for within-facility correlation of data. All analyses used Stata version 12. Key covariates, including distance to facility, duration on ART, HIV disclosure status to partner, parity, number of ANC visits, place of delivery, marital status, education, wealth quintile, age, region, health facility level, whether the health facility offers cervical cancer screening, whether the woman had postnatal care and knows what cervical cancer screening is, the frequency of screening, whether she knows any place where cervical cancer screening is conducted, and the perceived risk of getting cervical cancer, were included in the model for adjusted analyses.

## Results

Overall, 5198 women were interviewed, slightly over 1000 women in each of the five regions. The majority of the women were married (59.2%) or in a relationship (26.4%). More than half (56.3%) had attained primary education while 14% had no formal education. Among the household possessions, nearly two-thirds (64.9%) had a radio while 80.9% had a mobile phone, ranging from 74.8% in Northern Uganda to 94.5% in Kampala (Table 1). Overall, 5022 (96.6%) of the women had been on ART for a median time of 3 years (interquartile range 2–6 years).

**Table 1.** Selected characteristics of 15–49-year-old human immunodeficiency virus (HIV)-positive women in care.

		Region				
Characteristic	Total (*n* = 5198)	Kampala (*n* = 1048)	Central (*n* = 1032)	Eastern (*n* = 1034)	Western (*n* = 1039)	Northern (*n* = 1045)
Age (years)						
15–19	103 (2.0)	14 (1.3)	22 (2.1)	15 (1.5)	25 (2.4)	27 (2.6)
20–24	657 (12.6)	150 (14.3)	132 (12.8)	115 (11.1)	147 (14.1)	113 (10.8)
25–29	1147 (22.1)	273 (26.0)	209 (20.3)	215 (20.8)	238 (22.9)	212 (20.3)
30–34	1294 (24.9)	276 (26.3)	243 (23.5)	256 (24.8)	243 (23.4)	276 (26.4)
35–39	960 (18.5)	176 (16.8)	206 (20.0)	214 (20.7)	194 (18.7)	170 (16.3)
≥ 40	1037 (20.0)	159 (15.2)	220 (21.3)	219 (21.2)	192 (18.5)	247 (23.6)
Health facility level						
Hospital	1556 (29.9)	224 (21.4)	316 (30.6)	284 (27.5)	343 (33.0)	389 (37.2)
HCIV	1540 (29.6)	85 (8.1)	273 (26.5)	405 (39.2)	400 (38.5)	377 (36.1)
HCIII	1542 (29.7)	417 (39.8)	366 (35.5)	244 (23.6)	256 (24.6)	259 (24.8)
HCII	416 (8.0)	288 (27.5)	1 (0.1)	81 (7.8)	38 (3.7)	8 (0.8)
Private health unit	112 (2.2)	34 (3.2)	58 (5.6)	20 (1.9)	0 (0.0)	0 (0.0)
Other	32 (0.6)	0 (0.0)	18 (1.7)	0 (0.0)	2 (0.2)	12 (1.1)
Religion						
Catholic	2136 (41.1)	354 (33.8)	441 (42.7)	315 (30.5)	415 (39.9)	611 (58.5)
Anglican/Protestant	1616 (31.1)	284 (27.1)	273 (26.5)	370 (35.8)	410 (39.5)	279 (26.7)
Muslim	663 (12.8)	199 (19.0)	150 (14.5)	183 (17.7)	53 (5.1)	78 (7.5)
Pentecostal/Born Again/Evangelical	662 (12.7)	188 (17.9)	137 (13.3)	155 (15)	115 (11.1)	67 (6.4)
Other	121 (2.3)	23 (2.1)	31 (3.0)	11 (1.1)	46 (4.5)	10 (1.0)
Marital status						
Never married	107 (2.1)	25 (2.4)	7 (0.7)	23 (2.2)	32 (3.1)	20 (1.9)
In relationship but not married	1371 (26.4)	349 (33.3)	362 (35.1)	172 (16.6)	163 (15.7)	325 (31.1)
Married	3079 (59.2)	596 (56.9)	555 (53.8)	690 (66.7)	672 (64.7)	566 (54.2)
Divorced/separated	412 (7.9)	60 (5.7)	77 (7.5)	85 (8.2)	105 (10.1)	85 (8.1)
Widowed	229 (4.4)	18 (1.7)	31 (3)	64 (6.2)	67 (6.4)	49 (4.7)
Education^a^						
None	726 (14.0)	65 (6.2)	143 (13.9)	145 (14.0)	157 (15.1)	216 (20.7)
Primary	2924 (56.3)	449 (42.8)	606 (58.7)	565 (54.6)	653 (62.8)	651 (62.3)
Secondary	1381 (26.6)	445 (42.5)	260 (25.2)	303 (29.3)	221 (21.3)	152 (14.5)
More than secondary	156 (3.0)	89 (8.5)	21 (2)	21 (2.0)	7 (0.7)	18 (1.7)
Missing	11 (0.2)	0 (0.0)	2 (0.2)	0 (0.0)	1 (0.1)	8 (0.8)
Wealth quintile						
Lowest	1054 (20.3)	22 (2.1)	170 (16.5)	282 (27.3)	230 (22.1)	350 (33.5)
Second	1026 (19.7)	35 (3.3)	221 (21.4)	244 (23.6)	224 (21.6)	302 (28.9)
Middle	1041 (20.0)	64 (6.1)	231 (22.4)	253 (24.5)	249 (24.0)	244 (23.3)
Fourth	1039 (20.0)	328 (31.3)	228 (22.1)	146 (14.1)	239 (23.0)	98 (9.4)
Highest	1038 (20.0)	599 (57.2)	182 (17.6)	109 (10.5)	97 (9.3)	51 (4.9)
Owns a radio						
No	1822 (35.1)	346 (33.0)	294 (28.5)	399 (38.6)	354 (34.1)	429 (41.1)
Yes	3376 (64.9)	702 (67.0)	738 (71.5)	635 (61.4)	685 (65.9)	616 (58.9)
Owns a television						
No	3840 (73.9)	332 (31.7)	764 (74.0)	894 (86.5)	894 (86.0)	956 (91.5)
Yes	1358 (26.1)	716 (68.3)	268 (26.0)	140 (13.5)	145 (14.0)	89 (8.5)
Owns a mobile phone						
No	991 (19.1)	58 (5.5)	187 (18.1)	240 (23.2)	243 (23.4)	263 (25.2)
Yes	4207 (80.9)	990 (94.5)	845 (81.9)	794 (76.8)	796 (76.6)	782 (74.8)
Owns a regular landline						
No	5142 (98.9)	1031 (98.4)	1019 (98.7)	1022 (98.8)	1037 (99.8)	1033 (98.9)
Yes	56 (1.1)	17 (1.6)	13 (1.3)	12 (1.2)	2 (0.2)	12 (1.1)
Owns a bicycle						
No	3583 (68.9)	950 (90.6)	735 (71.2)	610 (59.0)	794 (76.4)	494 (47.3)
Yes	1615 (31.1)	98 (9.4)	297 (28.8)	424 (41.0)	245 (23.6)	551 (52.7)
Proximity to health facility (km)						
1–4	2121 (40.8)	503 (48.0)	423 (41.0)	430 (41.6)	358 (34.5)	407 (38.9)
5–9	1267 (24.4)	243 (23.2)	233 (22.6)	258 (25.0)	220 (21.2)	313 (30.0)
10–15	601 (11.6)	111 (10.6)	104 (10.1)	101 (9.8)	147 (14.1)	138 (13.2)
≥ 15	1209 (23.3)	191 (18.2)	272 (26.4)	245 (23.7)	314 (30.2)	187 (17.9)
On ART						
No	176 (3.4)	51 (4.9)	6 (0.6)	26 (2.5)	52 (5.0)	41 (3.9)
Yes	5022 (96.6)	997 (95.1)	1026 (99.4)	1008 (97.5)	987 (95.0)	1004 (96.1)
Duration on ART (years)						
Not on ART	205 (4.0)	57 (5.4)	11 (1.1)	34 (3.3)	58 (5.6)	52 (5.0)
< 1	551 (10.7)	166 (15.8)	128 (12.4)	87 (8.4)	100 (9.6)	78 (7.5)
< 2	566 (11.0)	127 (12.1)	129 (12.5)	105 (10.2)	110 (10.6)	99 (9.5)
≥ 2	3831 (74.4)	698 (66.6)	764 (74.0)	808 (78.1)	771 (74.2)	816 (78.1)
Disclosed HIV status to partner						
No	800 (15.5)	301 (29.0)	214 (20.8)	114 (11.1)	133 (12.9)	38 (3.7)
Yes	4362 (84.5)	736 (71.0)	813 (79.2)	914 (88.9)	902 (87.1)	997 (96.3)
No. of biological children						
0	1342 (25.8)	267 (25.5)	318 (30.8)	207 (20.0)	330 (31.8)	220 (21.1)
1	588 (11.3)	193 (18.4)	104 (10.1)	93 (9.0)	104 (10.0)	94 (9.0)
2	852 (16.4)	220 (21.0)	164 (15.9)	133 (12.9)	176 (16.9)	159 (15.2)
3	764 (14.7)	169 (16.1)	132 (12.8)	157 (15.2)	147 (14.1)	159 (15.2)
≥ 4	1652 (31.8)	199 (19.0)	314 (30.4)	444 (42.9)	282 (27.1)	413 (39.5)
Visited ANC at least once						
No	437 (9.2)	167 (17.5)	128 (13.5)	35 (3.8)	67 (7.0)	40 (4.2)
Yes	4304 (90.8)	786 (82.5)	817 (86.5)	889 (96.2)	895 (93.0)	917 (95.8)
ANC visits						
0	372 (8.1)	98 (11.6)	90 (10.2)	74 (7.8)	62 (6.5)	48 (5.0)
1–3	1334 (29.0)	235 (27.8)	256 (28.9)	273 (28.6)	258 (27.1)	312 (32.6)
≥ 4	2887 (62.9)	512 (60.6)	540 (60.9)	606 (63.6)	633 (66.4)	596 (62.3)
Place of delivery						
Elsewhere	223 (9.3)	25 (5.8)	60 (14.1)	52 (9.8)	50 (9.4)	36 (7.4)
Health facility	2184 (90.7)	406 (94.2)	365 (85.9)	481 (90.2)	481 (90.6)	451 (92.6)

Data are shown as *n* (%).

^a^Education categories refer to the highest level of education attended, whether or not that level was completed.

HC, health center; ART, antiretroviral therapy; ANC, antenatal care.

### Knowledge of cervical cancer and screening

Table 2 shows the women’s cervical cancer screening knowledge. Overall, 94.0% (*n *= 4858) had ever heard of cervical cancer screening. Knowledge of a site where they could go for cervical screening was 76.6% (*n *= 3732). When asked about how frequently screening should be done among HIV-infected women, 47.4% (*n *= 2302) did not know; this was highest in the Central region (69.0%; *n *= 664) and Kampala (52.1%; *n *= 531), among the uneducated women (51.9%; *n *= 342), and the 15–19-year-olds (55.8%; *n *= 48). However, 43.7% (*n *= 2123) mentioned screening at least once a year, and this was highest in the Eastern region (60.4%; *n *= 569). Half of the women (50%; *n *= 2409) did not know the signs of cervical cancer. Among those who knew, the most commonly cited symptoms were foul discharge (24.0%; *n *= 1146), heavy bleeding (19.0%; *n *= 903), and painful intercourse (14.0%; *n *= 673). In relation to the causes of cervical cancer, 22.6% (*n *= 1075) mentioned sexually transmitted diseases and 26.6% (*n *= 1266) multiple sexual partners; 38.6% (*n *= 1837) did not know the cause and 8.3% (*n *= 395) mentioned contraceptives as a cause of cervical cancer. Nearly half of the women (45.8%; *n *= 2336) said that cervical cancer is preventable while 20.9 (*n *= 1066) said that it was curable.

**Table 2.** Knowledge of cervical cancer screening.

Characteristic	Women who knew that cervical cancer screening should happen at least once a year	Women who knew place where cervical cancer screening is done
All women	5168 (40.9)	4872 (76.6)
Age (years)		
15–24	760 (35.1)	680 (66.8)
15–19	103 (28.2)	87 (64.4)
20–24	657 (36.2)	593 (67.1)
25–29	1147 (38.2)	1091 (75.8)
30–34	1294 (42.7)	1222 (77.8)
35–39	960 (43.9)	904 (79.6)
≥ 40	1037 (43.0)	975 (79.8)
Region		
Kampala	1034 (55.0)	1025 (77.5)
Central	1048 (42.6)	972 (68.9)
Eastern	1045 (50.0)	936 (75.9)
Western	1039 (37.2)	967 (76.6)
Northern	1032 (19.3)	972 (83.8)
Duration on ART (years)		
Not on ART	212 (35.4)	185 (74.1)
< 1	559 (32.4)	517 (66.9)
< 2	570 (33.9)	523 (69.8)
≥ 2	3857 (43.4)	3647 (79.0)
Marital status		
Never married	107 (37.4)	97 (69.1)
In relationship but not married	1371 (39.5)	1279 (76.9)
Married	3079 (42.2)	2912 (76.9)
Divorced/separated	412 (35.2)	371 (72.8)
Widowed	229 (43.2)	213 (80.3)
Education		
No education	726 (34.7)	662 (70.5)
Primary	2924 (39.4)	2714 (75.4)
Secondary	1381 (45.7)	1331 (81.1)
More than secondary	156 (51.9)	154 (85.1)
Missing	11 (63.6)	11 (63.6)
Wealth quintile		
Lowest	1054 (39.8)	957 (75.2)
Second	1026 (40.6)	954 (75.7)
Middle	1041 (40.6)	967 (73.4)
Fourth	1039 (40.2)	988 (78.4)
Highest	1038 (43.1)	1006 (79.8)

Data are shown as *n* (%).

ART, antiretroviral therapy.

### Risk perception, uptake of cervical screening, and HPV vaccination

The perceived lifetime risk of getting cervical cancer was rated as low by 33.7% (*n *= 1719) of the women, while 16.8% (*n *= 857) rated the risk as high and 23.5% (*n *= 1199) were not sure (Table 3).

**Table 3.** Uptake of cervical cancer screening.

		Region				
Characteristic	Total (*n* = 5153)	Kampala (*n* = 1042)	Central (*n* = 1032)	Eastern (*n* = 1026)	Western (*n* = 1016)	Northern (*n* = 1037)
All women	5153 (30.3)	1042 (39.1)	1032 (18.6)	1026 (34.3)	1016 (26.9)	1037 (32.6)
Age (years)						
15–24	754 (16.1)	164 (14.6)	154 (14.9)	129 (18.6)	168 (11.9)	139 (21.6)
15–19	102 (9.8)	14 (0)	22 (9.1)	15 (20)	24 (0)	27 (18.5)
20–24	652 (17)	150 (16)	132 (15.9)	114 (18.4)	144 (13.9)	112 (22.3)
25–29	1136 (26.9)	269 (35.3)	209 (12.4)	213 (29.6)	234 (23.9)	211 (31.3)
30–34	1284 (32.6)	276 (40.9)	243 (19.3)	253 (30.8)	237 (32.5)	275 (37.5)
35–39	950 (36.7)	175 (50.3)	206 (20.4)	212 (42)	190 (33.7)	167 (39.5)
≥ 40	1029 (35.8)	158 (44.9)	220 (24.6)	219 (55.3)	187 (70.1)	245 (29.8)
Marital status						
Never married	106 (23.6)	24 (50)	7 (0)	23 (26.1)	32 (12.5)	20 (15)
In relationship but not married	1358 (27.5)	346 (37.3)	362 (17.7)	168 (33.3)	161 (19.3)	321 (29)
Married	3059 (31.8)	595 (39.5)	555 (19.6)	688 (34.3)	657 (28.9)	564 (36)
Divorced/separated	405 (30.6)	59 (42.4)	77 (16.9)	83 (32.5)	103 (32)	83 (31.3)
Widowed	225 (29.8)	18 (33.3)	31 (19.4)	64 (42.2)	63 (23.8)	49 (26.5)
Education						
No education	722 (23.8)	65 (38.5)	143 (12.6)	145 (20.7)	155 (23.2)	214 (29.4)
Primary	2894 (28.2)	446 (37)	606 (16.2)	558 (32.3)	637 (25.3)	647 (32.8)
Secondary	1371 (36.5)	442 (38.2)	260 (26.9)	302 (44.4)	217 (33.2)	150 (36.7)
More than secondary	155 (45.8)	89 (53.9)	21 (28.6)	21 (38.1)	6 (50)	18 (33.3)
Missing	11 (27.3)		2 (0)		1 (100)	8 (25)
Religion						
Catholic	2113 (30.5)	349 (38.7)	441 (17.0)	312 (37.5)	609 (35.0)	402 (25.9)
Anglican/Protestant	1607 (31.1)	284 (40.5)	273 (18.3)	369 (37.9)	274 (29.2)	407 (28.3)
Muslim	658 (26.8)	199 (37.7)	150 (19.3)	180 (20.0)	78 (21.8)	51 (37.3)
Protestant/Born Again	656 (33.1)	187 (40.6)	137 (22.6)	154 (37.7)	66 (36.4)	112 (25.0)
Other	119 (23.3)	23 (30.0)	32 (23.3)	11 (10.0)	10 (42.9)	44 (19.4)
Wealth quintile						
Lowest	1046 (25)	22 (18.2)	170 (11.2)	279 (30.5)	228 (18.9)	347 (32)
Second	1018 (29.4)	34 (32.4)	221 (17.2)	244 (34.4)	218 (30.7)	301 (32.9)
Middle	1029 (27.4)	64 (37.5)	231 (16.5)	253 (36)	239 (23.4)	242 (30.2)
Fourth	1026 (33.1)	326 (37.4)	228 (25)	142 (34.5)	234 (32.9)	96 (36.5)
Highest	1034 (36.7)	596 (41.3)	182 (22)	108 (39.8)	97 (30.9)	51 (39.2)
Distance to health facility (km)						
0–4	2108 (31.9)	502 (37.8)	423 (21.7)	429 (36.4)	351 (27.4)	403 (34.2)
5–9	1256 (29.9)	239 (41.8)	233 (18.9)	253 (36)	218 (26.6)	313 (26.2)
10–14	595 (31.1)	111 (45.9)	104 (13.5)	100 (34)	143 (28.7)	137 (32.8)
≥ 15	1151 (27.5)	187 (35.3)	265 (15.8)	235 (28.5)	295 (26.4)	169 (37.9)
On ART						
No	160 (23.8)	49 (18.4)	1 (100)	24 (41.7)	52 (23.1)	34 (17.6)
Yes	4983 (30.5)	993 (40.1)	1026 (18.6)	1001 (34.1)	964 (27.1)	999 (33)
Duration on ART (years)						
Not on ART	205 (23.4)	55 (20.0)	11 (9.1)	33 (63.6)	57 (21.1)	49 (24.5)
< 1	551 (18.3)	166 (19.3)	128 (12.5)	83 (80.7)	97 (19.6)	77 (23.4)
< 2	566 (23.1)	126 (31.8)	129 (17.8)	104 (79.8)	108 (15.7)	99 (30.3)
≥ 2	3831 (33.5)	695 (46.6)	764 (19.9)	806 (62.4)	754 (29.8)	812 (34.2)
Health facility level						
Hospital	1546 (27.7)	224 (33.9)	316 (17.1)	281 (34.2)	339 (27.7)	386 (28)
HCIV	1517 (28.9)	85 (45.9)	273 (22.7)	400 (31)	384 (21.6)	375 (34.7)
HCIII	1535 (28.5)	415 (37.1)	366 (12)	244 (29.9)	253 (30.8)	257 (34.6)
HCII	411 (47)	284 (43.3)	1 (0)	81 (60.5)	38 (47.4)	7 (42.9)
Private health unit	112 (47.3)	34 (44.1)	58 (48.3)	20 (50)	2 (0)	12 (66.7)
Other	26 (38.5)	–	18 (22.2)	–	–	–
Visited ANC at least once						
No	364 (26.1)	165 (35.8)	128 (19.5)	35 (31.4)	65 (21.5)	40 (35)
Yes	4249 (30.3)	782 (39.5)	817 (17.7)	886 (34.7)	875 (27.1)	910 (32)
ANC visits						
0	364 (26.1)	97 (29.9)	90 (15.6)	69 (29)	60 (23.3)	48 (37.5)
1–3	1322 (25.5)	233 (33.9)	256 (16.8)	272 (25)	252 (20.6)	309 (30.7)
> 4	2866 (32.8)	511 (43.1)	540 (18.5)	604 (39.4)	619 (29.9)	592 (33.1)
DK/DR	61 (18)	34 (26.5)	17 (11.8)	2 (0)		8 (0)
Disclosed HIV status to partner						
Yes	4326 (31.6)	734 (41.8)	813 (19.6)	907 (36.7)	881 (27.8)	991 (32.8)
No	795 (23)	299 (32.4)	214 (15.4)	113 (15)	131 (20.6)	38 (23.7)
No. of biological children						
0	1327 (26.1)	265 (34.3)	318 (15.4)	205 (33.7)	322 (23.3)	217 (28.6)
1	583 (25.7)	191 (31.4)	104 (14.4)	93 (24.7)	102 (27.5)	93 (25.8)
2	846 (32.7)	219 (42.5)	164 (19.5)	132 (41.7)	172 (21.5)	159 (37.7)
3	760 (35)	168 (41.7)	132 (22)	156 (39.7)	146 (34.2)	158 (34.8)
4	668 (31.7)	112 (46.4)	141 (20.6)	149 (34.9)	112 (31.3)	154 (28.6)
5	433 (35.3)	52 (46.2)	75 (24)	120 (33.3)	65 (32.3)	121 (41.3)
≥ 6	536 (29.5)	35 (48.6)	98 (20.4)	171 (29.8)	97 (27.8)	135 (31.9)
Place of delivery						
Elsewhere	221 (15.84)	25 (16)	60 (10)	52 (21.2)	48 (10.4)	36 (25)
Health facility	2167 (28.6)	405 (35.6)	365 (18.4)	479 (32.6)	469 (26)	449 (29.2)
Had postnatal check-up						
No	989 (22.5)	202 (27.7)	152 (17.1)	209 (19.1)	271 (22.1)	155 (26.5)
Yes	208 (30.8)	15 (60)	22 (9.1)	65 (46.2)	33 (9.1)	73 (27.4)
DK/DR	28 (42.9)	1 (100)	3 (33.3)	7 (42.9)	5 (40)	12 (41.7)
Knowledge of need to screen for cervical cancer at least once a year						
No	3043 (16.3)	597 (19.8)	833 (13.7)	460 (15.2)	638 (16.5)	515 (17.3)
Yes	2110 (50.5)	445 (64.9)	199 (39.2)	566 (49.8)	378 (44.4)	522 (47.7)
Know any place where cervical cancer screening is done						
No	1132 (3.5)	252 (6)	362 (3)	318 (4.7)	289 (3.5)	225 (1.3)
Yes	3707 (40.7)	790 (49.6)	670 (27)	708 (47.6)	727 (36.2)	812 (41.3)
Likelihood of getting cervical cancer						
Low	1711 (39.7)	353 (49.3)	267 (26.2)	431 (45.7)	303 (31.7)	357 (39.8)
Medium	1315 (30.3)	189 (39.7)	190 (14.2)	221 (31.2)	361 (32.7)	354 (30.8)
High	850 (32.9)	190 (44.2)	198 (23.2)	195 (31.8)	108 (23.1)	159 (39.6)
DK	1188 (15.3)	310 (23.9)	377 (13)	179 (13.4)	244 (13.9)	167 (14.4)

Data are shown as *n* (%).

ART, antiretroviral therapy; HC, health center; ANC, antenatal care; DK, don’t know; DR, don’t remember; HIV, human immunodeficiency virus.

Uptake of cervical screening (having been screened at least once) was 30.3% (*n *= 1561) overall, highest in Kampala (39.1%; *n *= 407) and lowest in the Central region (18.6%; *n *= 192) (Table 3). Cervical cancer screening was also lower among the uneducated women (23.8%; *n *= 172) and those in the lowest wealth quartile (25.0%; *n *= 262). Among those who had ever been screened, about half (53.4%; *n *= 822) had been screened within the past year while 31.0% (482) had been screened 1–2 years before the interview. Most of the women had been screened only once (63.6%; *n *= 993), while 22.2% (*n *= 347) had been screened twice. The women who had been screened reported a number of negative experiences, including pain during the procedure (33.0%; *n *= 504), embarrassment (19.0%; *n *= 290), and discomfort (26.0%; *n *= 395). A painful procedure was reported by 34.5% (*n *= 334) of those undergoing screening for the first time, 29% (*n *= 98) of those undergoing screening for the second time, and 30.8% (*n *= 49) of those who had attended screening three or more times. Overall, 19.1% (*n *= 185) of the women undergoing screening for the time and 17% (*n *= 27) of those who had been screened three or more times felt that the screening procedure was embarrassing. However, 94.8% (*n *= 1437) of those who had ever been screened said they would honor subsequent screening schedules.

**Figure 1.**
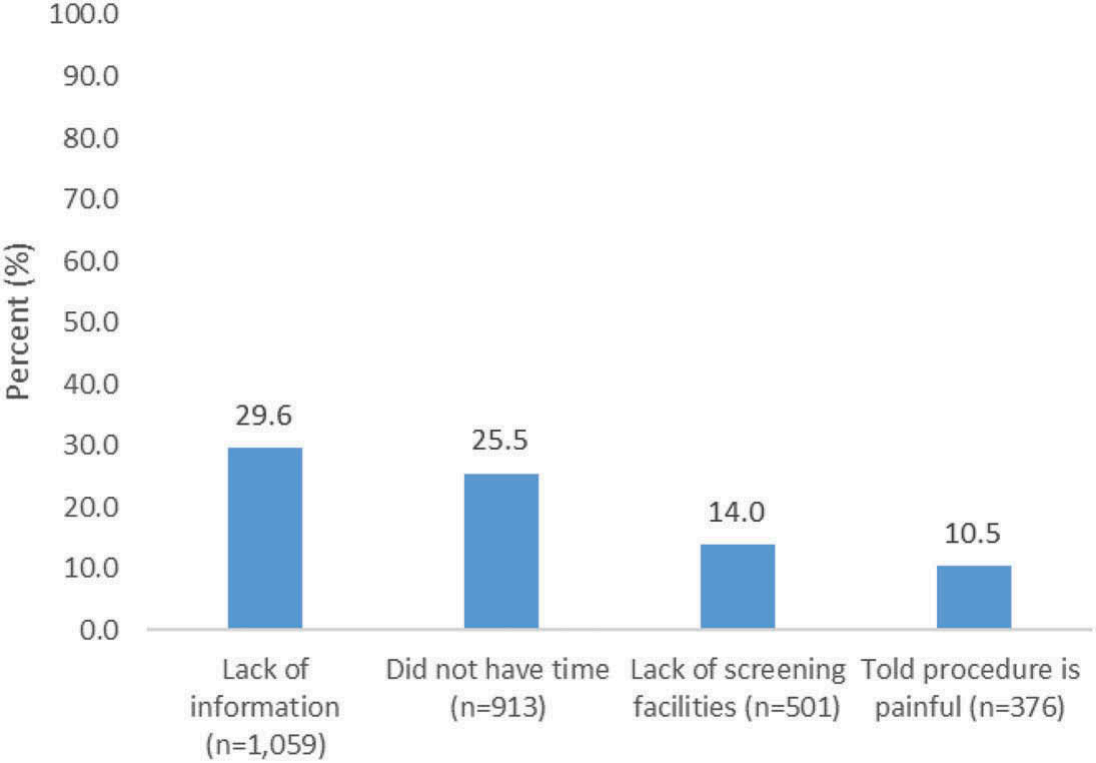
Reasons for not undergoing screening among women aged 15–49 years in HIV care, who had never been screened.

Among those who had never been screened, the most common reasons included lack of information (29.6%; *n *= 1059), lack of time (25.5%; *n *= 913), and lack of screening facilities (14.0%; *n *= 501). On the other hand, 10.5% (*n *= 376) of the women did not attend screening because they had been told that the procedure was painful (Figure 1). Fear of a painful procedure as a hindrance to screening was most common in the Central region (15.4; *n *= 128), which also had the lowest knowledge and screening uptake, and in the highest wealth quartile (14.1%; *n *= 91). When asked about the general barriers to screening in their community, a large proportion of the women mentioned fear of receiving a cancer diagnosis (40.6%; *n *= 1904), 35.1% (*n *= 1659) felt that there was a need for more information, 27.5% (*n *= 1281) mentioned awareness of services, and 22.3% (*n *= 1036) felt that the screening was embarrassing.

Uptake of cervical screening did not vary much with the distance to the health facility. However, the uptake increased with the number of biological children, and was higher among those who had attended four or more ANC visits and those who delivered at health facilities, as well as those who were on ART (Table 3). Based on the facility checklist, the majority of health facilities (64%; *n *= 153) offered cervical cancer screening, including the two national referral hospitals, 83% (*n *= 38) of the other hospitals, 62% (*n *= 47) of HCIV, 42% (*n *= 29) of HCIII, and 71% (*n *= 12) of HCII.

The overall prevalence of HPV vaccination was very low at 2% (95/5153), while among adolescents 15–19 years, eight of 102 (8%) had received HPV vaccination. Among the girls who had attained secondary level education, five of 27 (19%) had been vaccinated while two of 22 girls in the Central region had been vaccinated. None of the girls in the Western region reported HPV vaccination.

### Correlates of cervical cancer screening

On bivariate analysis, cervical cancer screening increased with the number of biological children and the number of years on ART. Women who delivered at health facilities, those who had secondary level education or higher, and those aged 24 years or older were more likely to have been screened. Compared to those in hospitals, women in HCII and private facilities were more likely to have been screened for cervical cancer. Women with knowledge of cervical cancer screening and where to attend screening were also more likely to have been screened. However, women in the Central and Western regions were less likely to have been screened (Table 4).

**Table 4.** Factors associated with testing for cervical cancer.

	Bivariate		Multivariate	
Characteristic	PR (95% CI)	*p*	PR (95% CI)	*p*
Distance to health facility (km)				
0–4	Ref			
≥ 5	1.09 (1.00–1.19)	0.041	1.028 (0.85–1.24)	0.770
Duration on ART (years)				
Not on ART	Ref			
< 1	0.78 (0.578–1.060)	0.114	0.82 (0.23–2.89)	0.752
< 2	0.99 (0.74–1.32)	0.937	1.16 (0.35–3.86)	0.808
≥ 2	1.43 (1.11–1.83)	0.005	1.31 (0.41–4.23)	0.652
ART				
No	Ref			
Yes	1.29 (0.97–1.70)	0.080	Not included	
Disclosed HIV status to partner				
No	Ref		Ref	
Yes	1.36 (1.20–1.57)	< 0.001	0.93 (0.67–1.29)	0.664
No. of biological children				
0	Ref		Ref	
1	0.99 (0.84–1.16)	0.874	0.52 (0.32–0.85)	0.009
2	1.26 (1.10–1.43)	0.001	0.68 (0.45–1.01)	0.056
3	1.34 (1.18–1.53)	< 0.001	0.67 (0.444–1.01)	0.053
≥ 4	1.26 (1.09–1.38)	0.001	0.71 (0.48–1.05)	0.084
Visited ANC at least once				
No	Ref			
Yes	1.063 (0.91–1.24)	0.446	Not included	
ANC visits				
0	Ref		Ref	
1–3	0.98 (0.80–1.19)	0.814	1.29 (0.20–8.10)	0.789
≥ 4	1.26 (1.05–1.50)	0.014	1.78 (0.29–11.10)	0.538
Place of delivery (among those who have ever delivered)				
Elsewhere	Ref		Ref	
Health facility	1.81 (1.32–2.47)	< 0.001	1.489 (0.903–2.456)	0.119
Currently married				
No	Ref		Ref	
Yes	1.13 (1.04–2.23)	< 0.001	1.22 (0.94–1.57)	0.131
Education				
None	Ref		Ref	
Primary	1.18 (1.03–1.37)	0.021	0.959 (0.701–1.313)	0.795
Secondary	1.53 (1.32–1.78)	< 0.001	1.022 (0.714–1.462)	0.906
Above secondary	1.92 (1.55–2.39)	< 0.001	0.838 (0.448–1.565)	0.578
Wealth quintile				
Lowest	Ref		Ref	
Second	1.17 (1.02–1.35)	0.028	1.12 (0.851–1.46)	0.429
Middle	1.09 (0.95–1.26)	0.222	0.92 (0.677–1.25)	0.590
Fourth	1.32 (1.16–1.52)	< 0.001	0.82 (0.595–1.13)	0.231
Highest	1.46 (1.282–1.67)	< 0.001	1.12 (0.800–1.57)	0.508
Age (years)				
15–19	Ref		Ref	
20–24	1.74 (0.94–3.20)	0.077	3.44 (0.56–21.27)	0.183
25–29	2.75 (1.51–4.99)	0.001	4.73 (0.76–29.18)	0.094
30–34	3.32 (1.83–6.01)	< 0.001	3.72 (0.60–23.01)	0.158
35–39	3.75 (2.07–6.79)	< 0.001	4.69 (0.76–28.98)	0.096
≥ 40	3.65 (2.01–6.61)	< 0.001	4.67 (0.73–29.75)	0.103
Religion				
Catholic	Ref			
Anglican/Protestant	1.02 (0.926–1.13)	0.68		
Muslim	0.88 (0.761–1.01)	0.07	Not included	
Protestant/Born Again	1.09 (0.956–1.23)	0.20		
Other	0.765 (0.535–1.092)	0.140		
Region				
Northern	Ref		Ref	
Kampala	1.20 (1.07–1.35)	0.002	1.07 (0.757–1.52)	0.695
Central	0.57 (0.49–0.67)	0.000	0.88 (0.596–1.30)	0.517
Eastern	1.05 (0.93–1.19)	0.410	0.88 (0.664–1.16)	0.360
Western	0.82 (0.72–0.94)	0.005	0.84 (0.621–1.13)	0.250
Health facility level				
Hospital	Ref		Ref	
HCIV	1.04 (0.932–1.17)	0.465	1.10 (0.85–1.41)	0.481
HCIII	1.03 (0.921–1.15)	0.600	1.16 (0.90–1.50)	0.254
HCII	1.70 (1.49–1.93)	0.000	1.89 (1.29–2.76)	0.001
Private health unit	1.709 (1.38–2.11)	0.000	1.93 (1.162–3.21)	0.011
Other	1.389 (0.85–2.27)	0.191	1.68 (1.09–2.87)	0.035
Had postnatal check-up				
No	Ref		Ref	
Yes	1.36 (1.08–1.73)	0.009	1.13 (0.9–1.41)	0.300
Knowledge of cervical cancer screening				
No	Ref		Ref	
Yes	3.10 (2.83–3.39)	< 0.001	2.19 (1.77–2.70)	< 0.001
Know any place where cervical cancer screening is done				
No	Ref			
Yes	11.51 (8.47–15.65)	< 0.001	6.47 (3.69–11.36)	< 0.001
Likelihood of getting cervical cancer				
High	Ref			
Low	1.21 (1.08–1.35)	0.001	1.52 (1.14–2.03)	0.004
Medium	0.919 (0.81–1.04)	0.189	1.19 (0.87–1.62)	0.270
DK	0.465 (0.40–0.55)	< 0.001	0.97 (0.67–1.39)	0.861

PR, prevalence ratio; CI, confidence interval; ART, antiretroviral therapy; HIV, human immunodeficiency virus; ANC, antenatal care; HC, health center; DK, don’t know.

Independent factors associated with screening, based on data obtained using modified Poisson regression, included the type of HIV facility they attended, knowledge of cervical cancer screening and place where they can attend screening, and their risk perception (Table 4). Screening was more common among women who attended the lowest level of health centers [HCII: adjusted (adj.) PR 1.89, 95% confidence interval (CI) 1.29–2.76] and private facilities (adj. PR 1.93, 95% CI 1.16–3.21) relative to public hospitals. Other factors associated with higher uptake of screening were knowledge that cervical screening should be carried out annually (adj. PR 2.16, 95% CI 1.77–2.70), knowledge of a place where screening is conducted (adj. PR 6.47, 95% CI 3.69–11.36), and low risk perception of cervical cancer (adj. PR 1.52, 95% CI 1.14–2.03).

## Discussion

This study assessed the uptake of cervical cancer screening and its correlates among a large number of HIV-infected women receiving HIV care from all five geographical regions of Uganda. Uptake of cervical screening was generally very low in this population even though it is known to be at high risk of cervical cancer, with only one-third of the women ever having been screened. More than three-quarters had been screened within 1–3 years preceding the study, which may indicate some recent efforts towards increasing screening. The study shows fairly large variations in uptake of screening: Kampala, the capital city, had twice the uptake of the Central region. Half of the women did not know the signs and symptoms of cervical cancer or the schedule for screening, while 36% did not know where to attend screening. These findings are similar to those from other studies conducted previously in Uganda and elsewhere in SSA [12].

Women had many fears and misconceptions related to cervical screening, which is of concern in a group at high risk of cervical cancer and calls for urgent interventions to integrate broader sexual and reproductive health services in HIV care. Several studies have previously documented widespread fears and misconceptions as a critical area for intervention [15,17]. The belief that contraceptives cause cervical cancer is detrimental to both cervical cancer prevention and family planning service efforts, and deserves attention.

The independent correlates of cervical screening revolved around the knowledge of screening, where to attend screening, and the level and type of health facility they attended. These factors highlight comprehensive knowledge as a critical demand factor and facility/service organization as an important supply factor that could enhance uptake of services if addressed. Logically, the women with high risk perception should have had higher uptake of cervical screening [19]. The women’s fears could explain the disconnect between risk perception and uptake of screening, since a significant proportion of women in the community indicated that they feared screening because they dreaded a diagnosis of cancer [17]. However, this study did not explore risk communication and perceived benefits of screening, which could potentially confound the relationship between risk perception and cervical screening [20,21].

These findings also highlight a number of missed opportunities for increasing cervical cancer screening among women in general, and among HIV-infected women in particular. Clearly, these women had interfaced with the health system for a long time but did not have sufficient knowledge and had not been screened. A large proportion of the women had attended ANC, delivered at health facilities, and been in HIV care for several years (median time on ART of 3 years). Furthermore, a significant proportion of those who had delivered at facilities and attended postnatal care had never been screened.

Mobile phones have been used to improve patient retention and adherence in several HIV services and could be extended to improving the uptake of other critical services. About 80% of the women had a mobile phone, a tool that could enhance education and be used to improve service uptake. Given the large number of women who require screening, investments in non-facility-based models of HPV screening, including community-based models, may improve efficiency [22,23].

Uptake of HPV vaccination among the young girls was extremely low, with virtually no girls having been vaccinated in some regions, which is a big missed opportunity for cervical cancer prevention among young girls given the high prevalence of HPV in this region [10,24,25]. The adolescent girls were also the least knowledgeable in relation to cervical cancer prevention and screening.

The majority of health facilities offered cervical cancer screening and, surprisingly, a larger proportion of the lower level HCII facilities provided screening than the higher level HCIII and HCIV. Women in private facilities were more likely to have been screened than those in public facilities, but the coverage was still very low overall. Noteworthy is the lack of a significant association between the provision of cervical cancer screening and uptake. This is likely to be overpowered by the influence of low knowledge and fear of screening for various reasons, and highlights the need for comprehensive education of women in facilities that have integrated cervical screening. The use of HIV clients, including ‘satisfied’ or ‘expert’ clients, in patient education and improvement of service uptake is a prominent feature of several HIV prevention and care interventions and could be extended to reproductive health services, including cervical screening.

### Limitations

This study does not fully explore why women who attended facilities that had cervical screening did not undergo screening and what prompted screening for those who did so, and may require follow-on qualitative studies and more comprehensive knowledge and perception measures, including the perceived benefits of screening. In addition, the cross-sectional nature of this study may limit the understanding of the associations. However, the study provides nationally representative data on knowledge and uptake of cervical screening and barriers to screening in a large number of HIV-infected women.

## Conclusion

In conclusion, this study highlights a very low uptake of cervical cancer screening in a very high-risk group, major knowledge gaps and misconceptions, and missed opportunities for cervical screening among women who interface with the health system. The findings call for urgent attention to be paid towards the integration of cervical screening into HIV prevention and care, and improvements in cervical cancer prevention efforts in general. Patient education to enhance comprehensive knowledge of cervical cancer screening and available support may increase the uptake of services.

## Paper context

Integration of cervical screening into HIV care is recommended but few large studies have explored service uptake. This study provides data on knowledge and uptake of cervical screening among 5198 HIV-infected women in care in all regions of Uganda. Overall, 47.4% (*n* = 2302) did not know the screening schedule and only 30.3% (*n* = 1561) had ever been screened, while HPV vaccination was 2% overall and 8% among adolescents. Interventions to enhance cervical screening and HPV uptake are urgently required.

## References

[1] World Health Organization 2014 Comprehensive cervical cancer control a guide to essential practice. [cited 2017 Jan 5] Available from: http://apps.who.int/iris/bitstream/10665/144785/1/9789241548953_eng.pdf 25642554

[2] De Vuyst H, Alemany L, Lacey C, et al The burden of human papillomavirus infections and related diseases in sub-saharan Africa. Vaccine. 2013;31: F32–F46. 24331746 10.1016/j.vaccine.2012.07.092 PMC4144870

[3] Huchko MJ, Maloba M, Nakalembe M, et al The time has come to make cervical cancer prevention an essential part of comprehensive sexual and reproductive health services for HIV-positive women in low-income countries. J Int AIDS Soc. 2015;18: 20282. 26643456 10.7448/IAS.18.6.20282 PMC4672400

[4] Coghill AE, Newcomb PA, Madeleine MM, et al Contribution of HIV infection to mortality among cancer patients in Uganda. AIDS. 2013;27: 2933–11. 23921614 10.1097/01.aids.0000433236.55937.cb PMC4319357

[5] Ngabo F, Franceschi S, Baussano I, et al Human papillomavirus infection in Rwanda at the moment of implementation of a national HPV vaccination programme. BMC Infect Dis. 2016;16: 225. 27221238 10.1186/s12879-016-1539-6 PMC4877733

[6] Thunga S, Andrews A, Ramapuram J, et al Cervical cytological abnormalities and human papilloma virus infection in women infected with HIV in Southern India. J Obstet Gynaecol Res. 2016;42:1822–1828. 27641071 10.1111/jog.13111

[7] Msyamboza KP, Phiri T, Sichali W, et al Cervical cancer screening uptake and challenges in Malawi from 2011 to 2015: retrospective cohort study. BMC Public Health. 2016;16: 806. 27535359 10.1186/s12889-016-3530-y PMC4989288

[8] Bansil P, Lim J, Byamugisha J, et al Performance of cervical cancer screening techniques in HIV-infected women in Uganda. J Low Genit Tract Dis. 2015;19:215–219. 25551591 10.1097/LGT.0000000000000090

[9] Tominski D, Katchanov J, Driesch D, et al The late-presenting HIV-infected patient 30 years after the introduction of HIV testing: spectrum of opportunistic diseases and missed opportunities for early diagnosis. HIV Med. 2016;1. 10.1111/hiv.12403 27478058

[10] Tathiah N, Naidoo M, Moodley I. Human papillomavirus (HPV) vaccination of adolescents in the South African private health sector: lessons from the HPV demonstration project in KwaZulu-Natal. S Afr Med J. 2015;105:954. 26937512 10.7196/samj.2015.v105i11.10135

[11] Campos NG, Tsu V, Jeronimo J, et al To expand coverage, or increase frequency: quantifying the tradeoffs between equity and efficiency facing cervical cancer screening programs in low-resource settings. Int J Cancer. 2016;7. 10.1002/ijc.30551 PMC5516173 27925175

[12] Ndejjo R, Mukama T, Musabyimana A, et al Uptake of cervical cancer screening and associated factors among women in rural Uganda: a cross sectional study. PLoS One. 2016;19;11(2):e0149696. 10.1371/journal.pone.0149696 PMC4760951 26894270

[13] Mwaka AD, Garimoi CO, Were EM, et al Social, demographic and healthcare factors associated with stage at diagnosis of cervical cancer: cross-sectional study in a tertiary hospital in Northern Uganda. BMJ Open. 2016;6: e007690. 10.1136/bmjopen-2015-007690 PMC4735146 26801459

[14] Mwaka AD, Orach CG, Were EM, et al Awareness of cervical cancer risk factors and symptoms: cross-sectional community survey in post-conflict northern Uganda. Health Expect. 2016;19:854–867. 26205470 10.1111/hex.12382 PMC4957614

[15] Hasahya OT1, Berggren V, Sematimba D, et al Beliefs, perceptions and health-seeking behaviours in relation to cervical cancer: a qualitative study among women in Uganda following completion of an HPV vaccination campaign. Glob Health Action. 2016;9: 29336. 10.3402/gha.v9.29336 PMC4759844 26895145

[16] Finocchario-Kessler S, Wexler C, Maloba M, et al Cervical cancer prevention and treatment research in Africa: a systematic review from a public health perspective. BMC Womens Health. 2016;16: 29. 27259656 10.1186/s12905-016-0306-6 PMC4893293

[17] Bukirwa A, Mutyoba JN, Mukasa BN, et al Motivations and barriers to cervical cancer screening among HIV infected women in HIV care: a qualitative study. BMC Womens Health. 2015;15: 82. 26458898 10.1186/s12905-015-0243-9 PMC4603977

[18] Kumakech E, Andersson S, Wabinga H, et al Integration of HIV and cervical cancer screening perceptions and preferences of communities in Uganda. BMC Womens Health. 2015;15:23. 25783655 10.1186/s12905-015-0183-4 PMC4359479

[19] Tilburt JC, James KM, Sinicrope PS, et al Factors influencing cancer risk perception in high risk populations: a systematic review. Hered Cancer Clin Pract. 2011;9:2. 21595959 10.1186/1897-4287-9-2 PMC3118965

[20] Bazargan M, Lucas-Wright A, Jones L, et al Understanding perceived benefit of early cancer detection: community-partnered research with African American women in South Los Angeles. J Womens Health (Larchmt). 2015;24:755–761. 26131760 10.1089/jwh.2014.5049 PMC4589099

[21] Hopwood P Breast cancer risk perception: what do we know and understand? Breast Cancer Res. 2000;2:387–391. 11250730 10.1186/bcr83 PMC138659

[22] Ogilvie GS, Mitchell S, Sekikubo M, et al Results of a community-based cervical cancer screening pilot project using human papillomavirus self-sampling in Kampala, Uganda. Int J Gynaecol Obstet. 2013;122:118–123. 23731506 10.1016/j.ijgo.2013.03.019

[23] Paul P, Winkler JL, Bartolini RM, et al Screen-and-treat approach to cervical cancer prevention using visual inspection with acetic acid and cryotherapy: experiences, perceptions, and beliefs from demonstration projects in Peru, Uganda, and Vietnam. Oncologist. 2013;18:1278–1284. 24217554 10.1634/theoncologist.2013-0253 PMC3868422

[24] Turiho AK, Muhwezi WW, Okello ES, et al Human papillomavirus (HPV) vaccination and adolescent girls’ knowledge and sexuality in Western Uganda: a comparative cross-sectional study. PLoS One. 2015;10:e0137094. 26327322 10.1371/journal.pone.0137094 PMC4556485

[25] Mugisha E, LaMontagne DS, Katahoire AR, et al Feasibility of delivering HPV vaccine to girls aged 10 to 15 years in Uganda. Afr Health Sci. 2015;15:33–41. 25834528 10.4314/ahs.v15i1.5 PMC4370128

